# Influence of Co doping on phase, structure and electrochemical properties of hydrothermally obtained Co_x_Zn_1−x_Fe_2_O_4_ (x = 0.0–0.4) nanoparticles

**DOI:** 10.1038/s41598-023-29830-3

**Published:** 2023-02-13

**Authors:** Thanin Putjuso, Sasitorn Putjuso, Attaphol Karaphun, Pairot Moontragoon, Isara Kotutha, Ekaphan Swatsitang

**Affiliations:** 1 Department of Physics and Mathematics, Faculty of Liberal Arts, Rajamangala University of Technology Rattanakosin, Wang Klai Kangwon Campus, Hua Hin, 77110 Prachuap Khiri Khan Thailand; 2 Institute of Nanomaterials Research and Innovation for Energy (IN-RIE), Khon Kaen University, Khon Kaen, 40002 Thailand; 3 Department of Physics, Faculty of Science, Khon Kaen University, Khon Kaen, 40002 Thailand; 4 Department of Applied Physics, Faculty of Engineering, Rajamangala University of Technology ISAN, Khon Kaen Campus, Khon Kaen, 40000 Thailand

**Keywords:** Energy science and technology, Materials science

## Abstract

In this work, Co_x_Zn_1−x_Fe_2_O_4_ (x = 0.0–0.4) nanoparticles (NPs) were successfully synthesized by a hydrothermal method at 200 °C for 12 h. X-ray diffraction revealed a pure cubic spinel phase of all samples with space group Fd-3m. Fourier transform infrared spectrometry disclosed the vibrational modes of metal oxides in the spinel structure. Scanning electron microscopy and transmission electron microscopy disclosed a uniform distribution of cuboidal shape NPs with a decreased average NPs size from 22.72 ± 0.62 to 20.85 ± 0.47 nm as the Co content increased. X-ray absorption near edge spectroscopy results confirmed the presence of Zn^2+^, Co^2+^ and Fe^2+^/Fe^3+^ in Co-doped samples. The pore volume, pore size and specific surface area were determined using N_2_ gas adsorption/desorption isotherms by the Brunauer–Emmett–Teller (BET) and Barrett–Joyner–Halenda (BJH) techniques. Electrochemical properties of supercapacitors, having active Co_x_Zn_1−x_Fe_2_O_4_ (x = 0.0–0.4) NPs as working electrodes, indicated pseudo-capacitor performance related to the Faradaic redox reaction. Interestingly, the highest specific capacitance (*Csc*), 855.33 F/g at 1 A/g, with a capacity retention of 90.41% after 1000 GCD cycle testing was achieved in the Co_0.3_Zn_0.7_Fe_2_O_4_ electrode.

## Introduction

Currently, multi-functional nanomaterials with intriguing properties are very important and necessary for many emerging novel technologies. They are essential parts of many electronic devices, specifically as electrodes in the energy collectors of fuel cells, batteries and supercapacitors (SCs). Moreover, increasing need for them is seen due to the immense increase in the demand for clean and sustainable energy. Particularly, SCs are a unique type of electrochemical device that stores energy via a collection of charges/ions on the electrodes surface, which can offer many benefits over conventional batteries and capacitors^1–3^. Generally, a SC cell consists of two separate electrodes immersed in an electrolyte. The chemical reactions between charges/ions in the electrolyte and electrodes materials are key functions that directly impact the performance of a SC cell. Practically, two important types of SCs are pseudo-capacitors (PCs) and electric double layer capacitors (EDLCs)^4,5^. Principally, each type of SC has similar cell structure, but a different charge storage mechanism and electrode material. For EDLCs, charges are stored in a very thin double layer on electrodes surface. Whereas in PCs, the reversible redox reactions are the major response for charge storage. In both types of SCs, the cell efficiency strongly depends on many parameters such as surface area, pore structure, and conductivity of active materials used for electrode fabrication^4^. Thus, a search for novel electrode materials with these good properties is an active area for researchers^6^. Usually, for PCs, various transition metal oxides (TMOs) and conducting polymers are generally employed for electrode fabrication because they can provide higher specific capacitance (*Csc*) and energy density (*E*_*d*_). This due to their fast Faradaic redox reaction during charging/discharging compared with carbon materials^7^. Moreover, numerous nanosized (5–50 nm) TMOs have been extensively studied. Many of them were found to be suitable for SCs electrodes with high electrochemical performance. This is due to their increased electroactive sites which offer appropriate conductive pathways and extensive nanopaths to promote efficient transportation of charges/ionic species^2,8–10^. During the past decade, spinel TMOs of the AB_2_O_4_ structure (A and B represent metal elements at tetrahedral and octahedral sites, respectively ) of CuFe_2_O_4_^2^, MnFe_2_O_4_^4^, NiFe_2_O_4_^3,6^, CoFe_2_O_4_^1,6,7,9^ and ZnFe_2_O_4_^10–15^ were extensively studied as electrode materials for PCs. This is due to their advantage of a polymorphic structure of normal, inverse and mixed spinel forms in which metal ions with a + 2 oxidation state (M^2+^) and (Fe^2+^/Fe^3+^) can be distributed at A or/and B sites. In normal spinel, M^2+^ and (Fe^2+^/Fe^3+^) ions are at A and B sites, respectively. For inverse spinel, M^2+^ ions replace some of (Fe^2+^/Fe^3+^) ions at the B site, while (Fe^2+^/Fe^3+^) ions can occupy both the A and B sites. In the case of mixed spinel, M^2+^ and (Fe^2+^/Fe^3+^) ions can be distributed at both A and B sites. As reported by earlier researchers, the *Csc* values of various AB_2_O_4_ ferrites of different morphologies vary over a wide range from a hundred to more than a thousand. For example, Ni_0.4_Co_0.6_Fe_2_O_4_ nanoparticles (NPs) (237 F/g at 1 A/g)^5^, CoFe_2_O_4_/NiFe_2_O_4_ nanospheres (269 F/g at 1 A/g)^6^, electrospun carbon/CuFe_2_O_4_ nanofibers (191 F/g at 10 mV/s)^2^, NiFe_2_O_4_@CoFe_2_O_4_ core–shell nanofibers (480 F/g at 1 A/g)^3^, ZnFe_2_O_4_ NPs (712 F/g at 2 mV/s)^15^, CoFe_2_O_4_ NPs (1,210 F/g at 1 F/g)^1^ and Ni_1−x_Mn_x_Fe_2_O_4_ NPs (1,221 F/g at 0.5 A/g)^4^. Among numerous AB_2_O_4_ materials, spinel zinc ferrite (ZnFe_2_O_4_) is of great interest. It has been extensively investigated due to its fascinating properties, natural richness, low cost, and environmental friendliness^10,11,15^. However, the development of high-performance ZnFe_2_O_4_ electrode materials remains a greater challenge due to their poor stability in cycling tests, as reported by Vadiyar et al*.*^12^. Owing to this problem, different approaches such as using material composites^13^ and structure modifications by doping with various transition metals (TMs) in M_x_Zn_1−x_Fe_2_O_4_ (for instance M = Co, Cu, Ni, Mn) have been attempted. However, doping with TMs was suggested as the effective method because it can significantly affect the ion distribution at A and B sites of spinel crystals of these materials and on the surface of ferrites. This makes them more active and sensitive to Faradaic redox reactions during charging/discharging process^16^. From literatures, Co was considered to be one of the effective TM that had been intensively used as dopant with successful improvement many properties of materials^17–19^.

Herein, it is our aim to synthesize Co_x_Zn_1−x_Fe_2_O_4_ (x = 0.0–0.4) NPs using a hydrothermal method, because it is a facile process that can generally occur at low temperature, without toxicity due to the use of metallic salts that can be dissolved in water^20,21^. Moreover, a high yield NPs of narrow size distribution can be obtained as compared to other method^22^. The influence of Co doping on the physical properties of the materials such as their phase, crystal structure, morphology, functional groups, surface area and oxidation states of metal ions, were studied using various techniques. Furthermore, the electrochemical properties of the obtained products were studied for SCs application. To the best of our knowledge, the electrochemical properties of hydrothermally obtained Co-doped ZnFe_2_O_4_ have never been reported.

## Experimental

### Material synthesis

**Figure 1:**
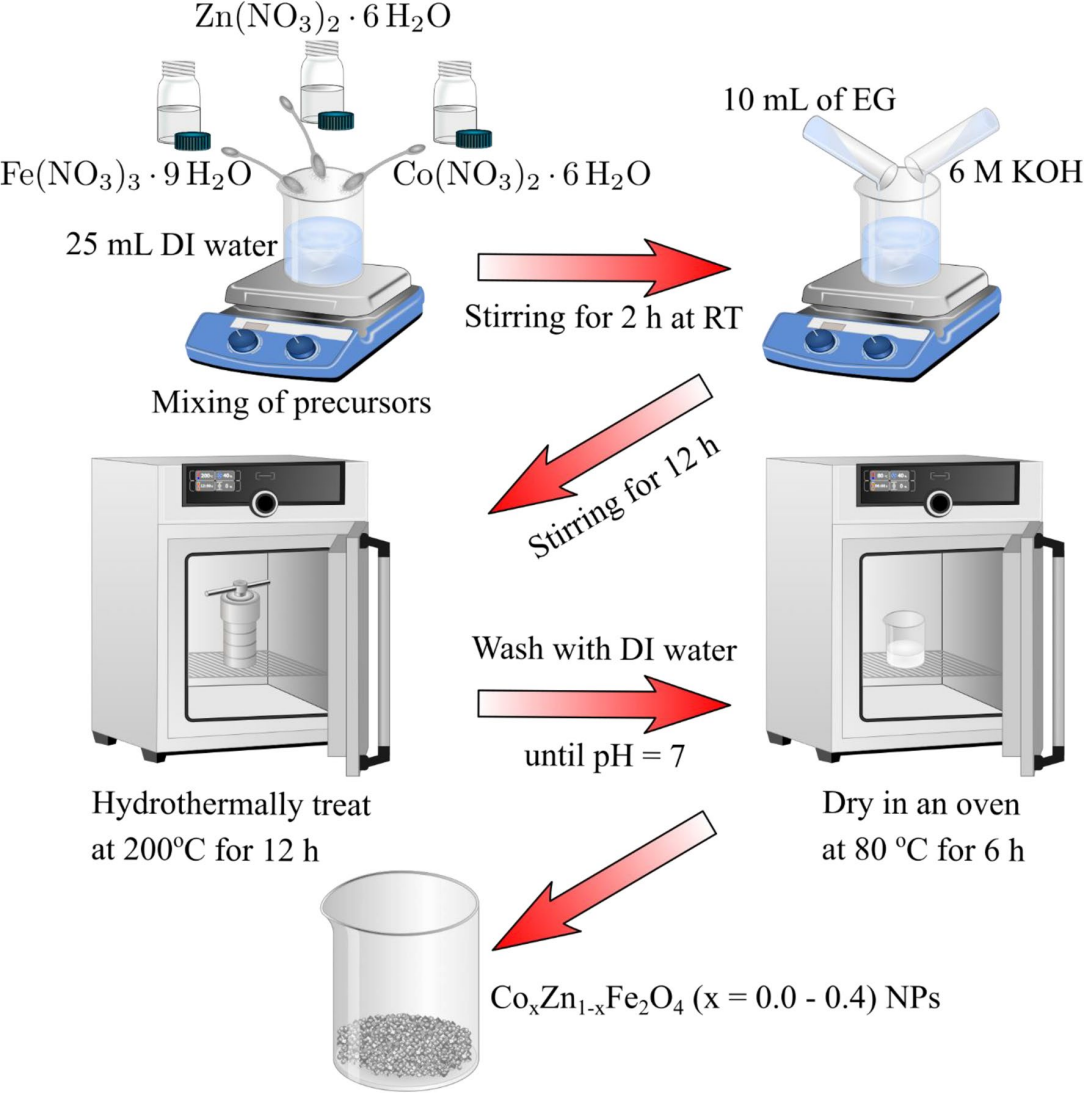
Synthesis of Co_x_Zn_1−x_Fe_2_O_4_ (x = 0.0–0.4) NPs by a hydrothermal method.

Co_x_Zn_1−x_Fe_2_O_4_ (x = 0.0–0.4) NPs were prepared by a hydrothermal method, as schematically shown in Fig. 1. Initially, the stoichiometric amounts of iron nitrate (Fe(NO_3_)_3_.9H_2_O, 5 mmol), zinc nitrate (Zn(NO_3_)_2_.6H_2_O, with 2.5-x mmol) and cobalt nitrate (Co(NO_3_)_2_.6H_2_O, with x = 0.0, 0.1, 0.2, 0.3 and 0.4 mmol) were dissolved in 25 mL deionized (DI) water, using a magnetic stirrer for 2 h at room temperature (RT). Subsequently, 10 ml ethylene glycol (EG) and 6 M KOH were added into the solution and magnetically stirred for 12 h. The obtained solution was hydrothermally treated in a tightly closed autoclave at 200 °C for 12 h in an oven (Memmert, UF55, USA). The obtained products were rinsed with DI water until they became neutral. Finally, the materials were dried in an oven at 80 °C for 6 h and ground to fine powders for further study.

### Characterization

The phase and crystal structure of Co_x_Zn_1−x_Fe_2_O_4_ (x = 0.0–0.4) NPs were characterized using X-ray diffraction (XRD, a Philips PW3040, Cu K_α_, $\lambda$ = 0.15406 nm). Transmission electron microscopy (TEM, JEOL2010) and scanning electron microscopy (SEM, M-SEM, SNE-4500) were performed to reveal the morphology and structure of the materials which were used to calculate particle size. Fourier transform infrared spectrophotometry (Bruker, Senterra) was used to verify the vibrational modes of bonding between metals and oxygen molecules in the crystal structure. The oxidation states of Zn, Co and Fe cations were analyzed using X-ray absorption near edge spectroscopy (XANES) performed at the K-edge in a transmission mode using the synchrotron light of Beamline 1.1W (2 GeV). Pore volume, pore size and specific surface area were investigated using N_2_ gas adsorption/desorption isotherms by the Brunauer–Emmett–Teller (BET) and Barrett–Joyner–Halenda (BJH) techniques (Autosorb-1, Quantachrome). A potentiostat/galvanostat station (CS350 electrochemical workstation; Corrtest, Hubei) was employed for electrochemical property studies via cyclic voltammetry (CV), galvanostatic charge–discharge (GCD) and electrochemical impedance spectroscopy (EIS) measurements in an aqueous 3 M KOH electrolyte using a three-electrode configuration.

### Electrode fabrication

The main parts of a cell for electrochemical measurements consists of a working electrode, having Co_x_Zn_1−x_Fe_2_O_4_ (x = 0.0–0.4) NPs as active materials with platinum wire and Ag/AgCl as counter and reference electrodes, respectively. CV testing was done at various scan rates (5–100 mV/s) in a potential range from −0.2 to 0.6 V. GCD measurements were performed at 1, 1.5, 2, 2.5, 3, 4, 5, 7.5 and 10 A/g. The stability of electrodes was evaluated after 1000 GCD cycle testing at 5 A/g. Specific capacitance (*Csc*) was determined using Eq. (1),^4^

1 $$Csc=\frac{(I\times (\Delta t))}{m\Delta V}$$

where *Csc* is obtained in F/g, *I* is the current density (A/g), *Δt* is the charging/discharging time (s), *m* and *ΔV* represent mass of active materials (g) and working potential window (V), respectively. The specific energy density (*E*_*d*_, Wh/kg) of electrode was directly related to the *Csc* value and evaluated using Eq. (2),

2 $${E}_{d}=\frac{1}{2}\frac{({C}_{sc,}\times (\Delta V{)}^{2})}{3.6}$$

In principle, the specific power density (*Pd,* W/kg) can be derived from *E*_*d*_ and discharge time *t* as shown in Eq. (3),

3 $${P}_{d}=\frac{{E}_{d}\times 3600}{t}$$

## Results and discussion

### Phase and structure analysis

**Figure 2:**
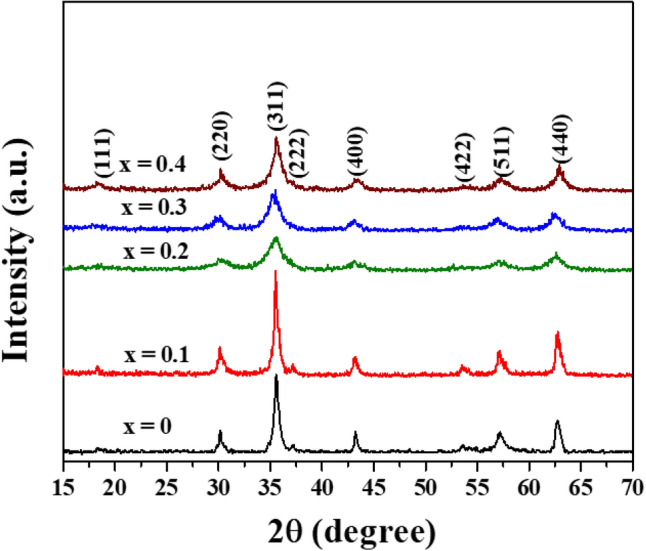
XRD patterns of Co_x_Zn_1−x_Fe_2_O_4_ (x = 0.0–0.4) NPs.

**Figure 3:**
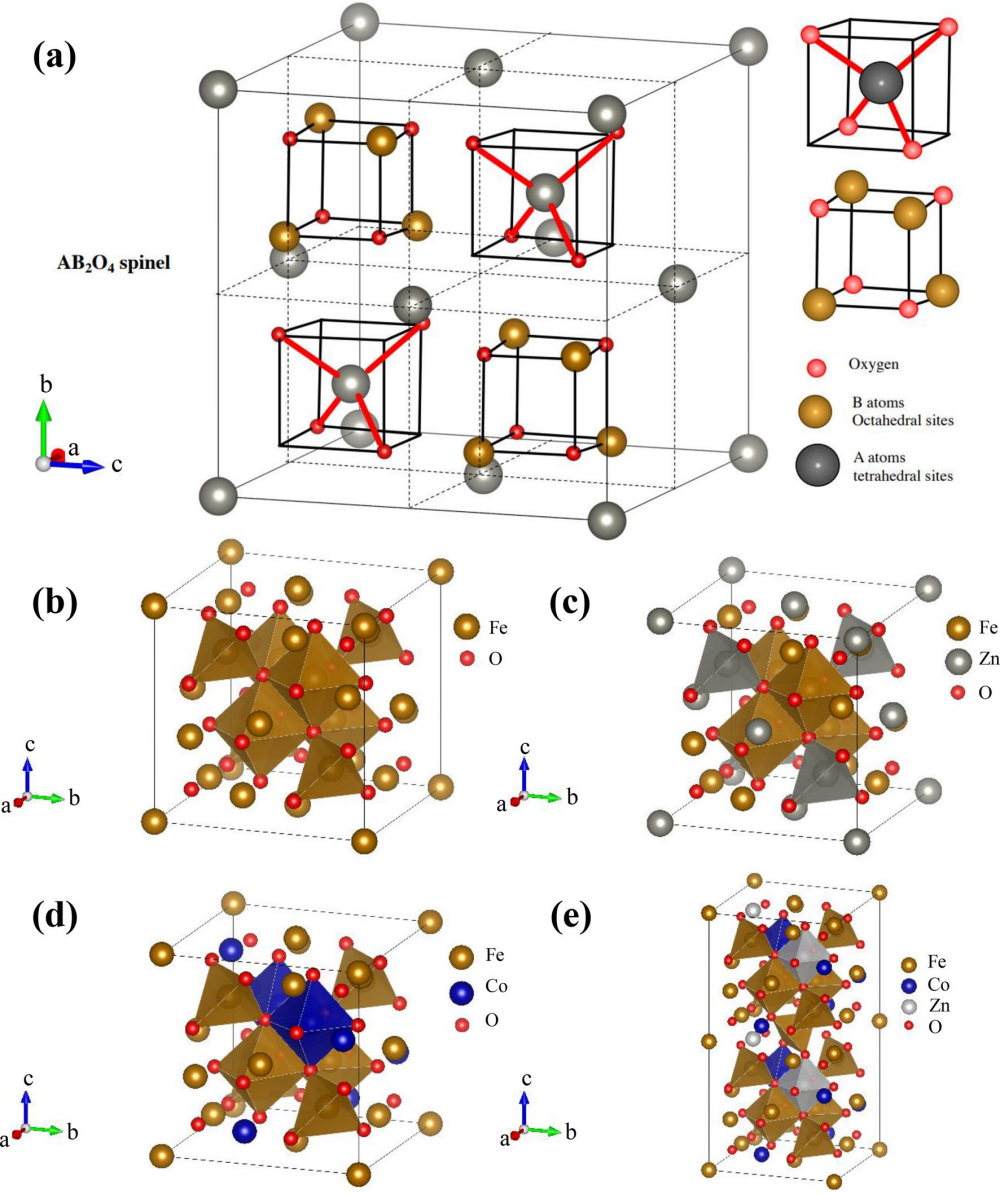
(**a**) Spinel model, showing A (tetrahedral) and B (octahedral) sites. Crystal structure of (**b**) Fe_3_O_4_, (**c**) normal spinel ZnFe_2_O_4_, (**d**) inverse spinel CoFe_2_O_4_ and (**e**) mixed spinel Co_0.3_Zn_0.7_Fe_2_O_4_ NPs.

**Figure 4:**
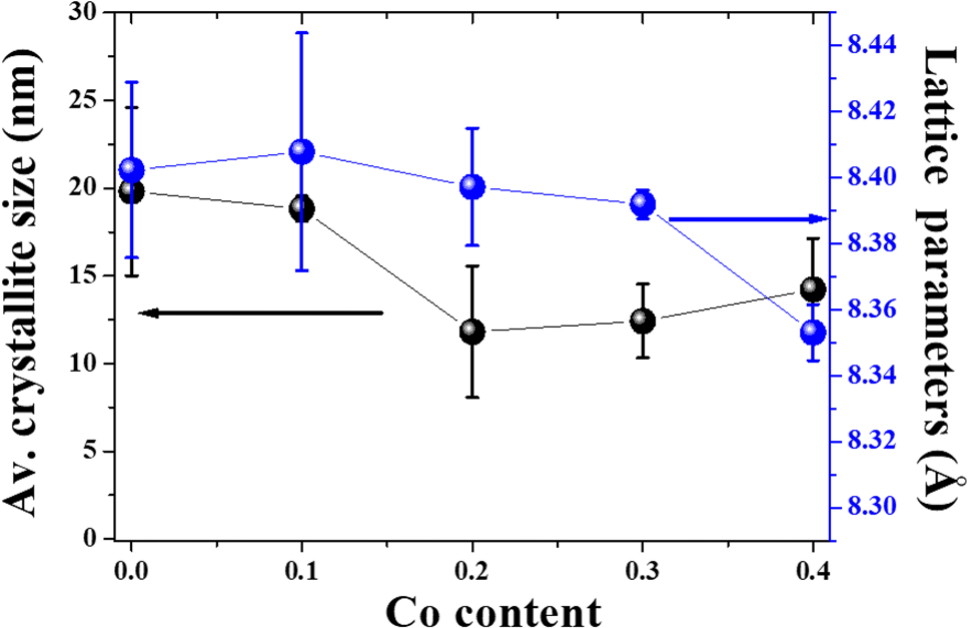
Plots of the lattice parameter (*a*) and Av. crystallite size (*D*_XRD_) of Co_x_Zn_1−x_Fe_2_O_4_ (x = 0.0–0.4) NPs as a function of Co content.

In Fig. 2, the XRD results of Co_x_Zn_1−x_Fe_2_O_4_ (x = 0.0–0.4) NPs in a 2$\theta$ range from 15° to 70° degree display well distinguishable crystalline peaks, consisting of the (111), (220), (311), (222), (400), (422), (511), and (440) planes. This matches the cubic spinel structure of crystalline ZnFe_2_O_4_ standard data (JCPDS Card No. 22–1012) within the Fd-3m space group, in agreement with the work of Perumal et al*.*^10^ and Mohamed et al*.*^18^. Moreover, all these XRD patterns correspond to a cubic spinel structure of crystalline Fe_3_O_4_ and CoFe_2_O_4_, as well, since these TMOs have the same crystalline structure shown in Fig. 3a–e.^23^ These structures illustrate a typical spinel model of Fe_3_O_4_, a normal spinel of ZnFe_2_O_4_, an inverse spinel of CoFe_2_O_4_ and a mixed spinel of Co_x_Zn_1−x_Fe_2_O_4_ ferrites, respectively. According to our previous study on the magnetic properties of hydrothermally synthesized Co_x_Zn_1−x_Fe_2_O_4_ ferrites^24^ and similar work of Gözüak et al*.*^25^, Mohamed et al*.*^18^, Mathew and Juang^26^ and Feng et al*.*^27^, it can be concluded that our hydrothermally synthesized Co_x_Zn_1−x_Fe_2_O_4_ (x = 0.1–0.4) NPs are ferrites of a mixed spinel structure. Moreover, the absence of impurities and other secondary phases confirms the purity of all samples and implies that Co ions can be successfully substituted for Zn ions in the ferrite structure. Furthermore, the lattice parameter (*a*) and average (Av.) crystallite size (*D*_XRD_) of all samples were determined by Rietveld refinement using the dominant (220), (311), (400), (422), (511), (440) reflection planes and Scherrer’s formula presented as Eq. (4):^7^

4 $$D_{{{\text{XRD}}}} = k\lambda /(\beta {\text{cos}}\theta ),$$

where the constant *k* is associated with crystalline shape and is generally taken as 0.9. *β* is the full width at half maximum of the involved diffraction peaks in radians and X-ray wavelength λ is in nanometers (nm). Calculated *a* and *D*_XRD_ values of Co_x_Zn_1−x_Fe_2_O_4_ NPs are tabulated in Table 1 and their plots versus Co content are shown in Fig. 4. In this figure, both *a* and *D*_XRD_ decrease with increasing Co content, in agreement with the work of Malik et al*.*^28^. Slight decreases in *a* and *D*_XRD_ with Co loading are subjected to substitution of the smaller ionic radii Co^2+^ ions (0.580 Å at A site, 0.745 Å at B site) on Zn^2+^ ions (0.600 Å at A site, 0.740 Å at B site), including the displacement and redistribution of Fe^2+^ ions (0.63 Å at A site, 0.78 Å at B site) and Fe^3+^ ions (0. 49 Å at A site, 0.645 Å at B site), as reported by Mohamed et al*.* (2019) in a study of Co_x_Zn_1−x_Fe_2_O_4_ NPs^18^. The oxidation states of Co, Zn and Fe ions will be further discussed with the XANES analysis below.

**Table 1:** Crystal structure, lattice parameter (*a*), Av. crystallite size (*D*_XRD_), Av. particle size determined by SEM and TEM of Co_x_Zn_1−x_Fe_2_O_4_ (x = 0.0–0.4) NPs.

Parameter	ZnFe_2_O_4_ NPs	Co_x_Zn_1−x_Fe_2_O_4_ NPs			
		x = 0.1	x = 0.2	x = 0.3	x = 0.4
Crystal structure	Cubic	Cubic	Cubic	Cubic	Cubic
Lattice parameter *a* (Å)	8.402	8.407	8.397	8.391	8.353
D_XRD_ (nm)	19.81 ± 4.8	18.82 ± 0.72	11.81 ± 3.76	12.43 ± 2.1	14.23 ± 2.9
Av. particle size by SEM (nm)	41.74 ± 0.22	40.67 ± 0.13	37.65 ± 0.11	38.61 ± 0.15	40.33 ± 0.13
Av. particle size by TEM (nm)	22.72 ± 0.62	21.85 ± 0.46	18.35 ± 0.32	20.12 ± 0.35	20.85 ± 0.47

### Morphological study

**Figure 5:**
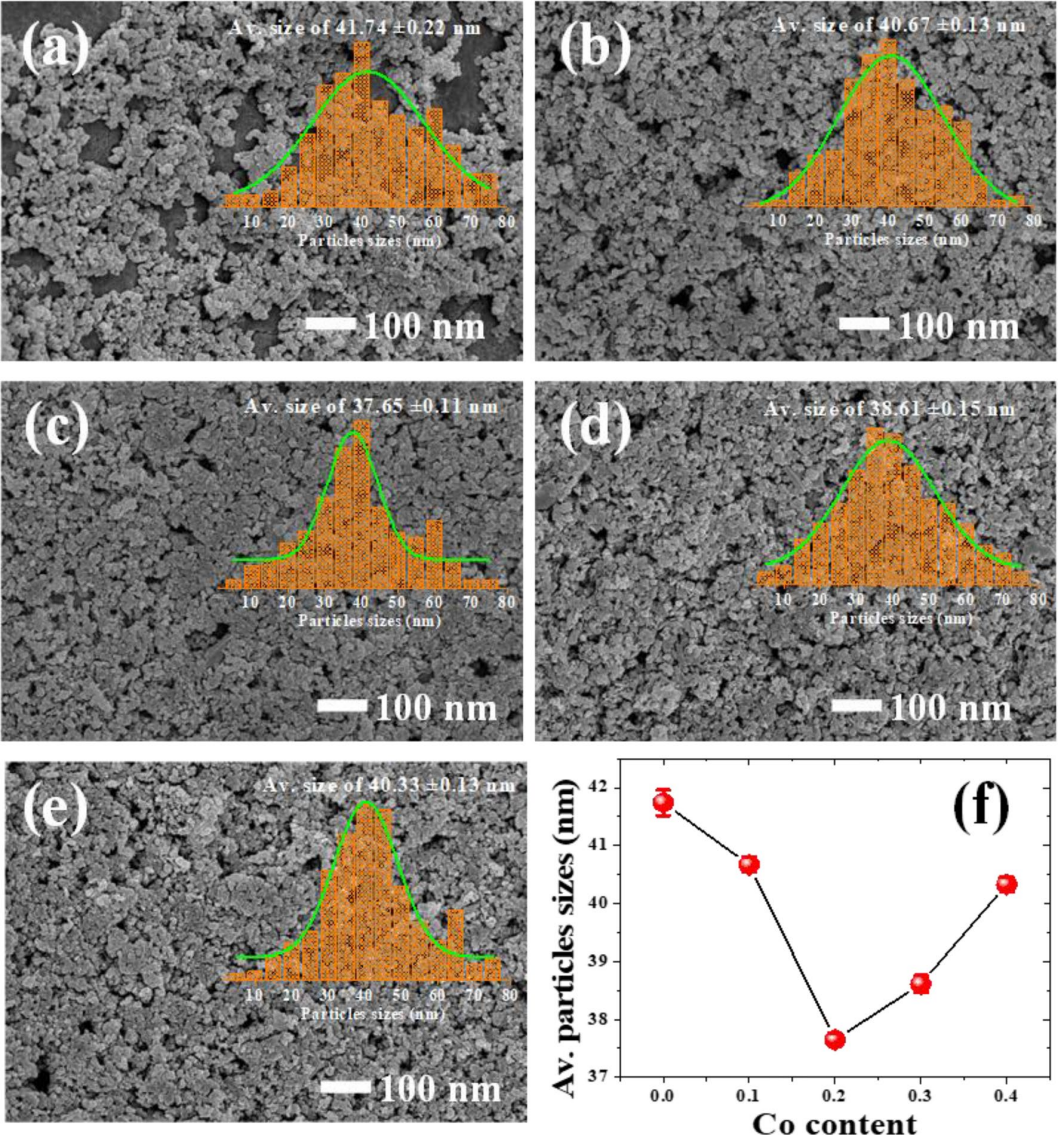
SEM images with histograms of the particle size distribution of Co_x_Zn_1−x_Fe_2_O_4_ NPs (**a**) x = 0.0, (**b**) x = 0.1, (**c**) x = 0.2, (**d**) x = 0.3 and (**e**) x = 0.4. (**f**) Plot of Av. particles sizes *vs*. Co content.

SEM micrographs of Co_x_Zn_1−x_Fe_2_O_4_ (x = 0.0–0.4) NPs in Fig. 5a–e show a uniform distribution of particles of a cuboidal shape. The particle sizes were estimated, and the obtained average (Av.) values are listed in Table 1. Moreover, plots of Av. particle size *vs.* Co content is also shown in Fig. 5f. As seen in Table 1 and the plot in Fig. 5f, the Av. nanometer particle sizes decrease from 41.74 ± 0.22 to 40.33 ± 0.13 nm with increasing Co content from x = 0.1–0.4. The decreased particle sizes of Co_1−x_Zn_x_Fe_2_O_4_ presented the lowest value for x = 0.2 and then increase with Co content (for x = 0.3 and 0.4). This might be dependent on different growth rates. Co cations may be substituted at A and B sites in different amounts during the preparation process. The homogeneous distribution of NPs was suggested to provide an enhanced active surface area with beneficial pathways for the electrolyte penetration and fast ion/electron transfer. This would result in enhanced electrochemical performance, as reported by Feng et al*.*^6^.

**Figure 6:**
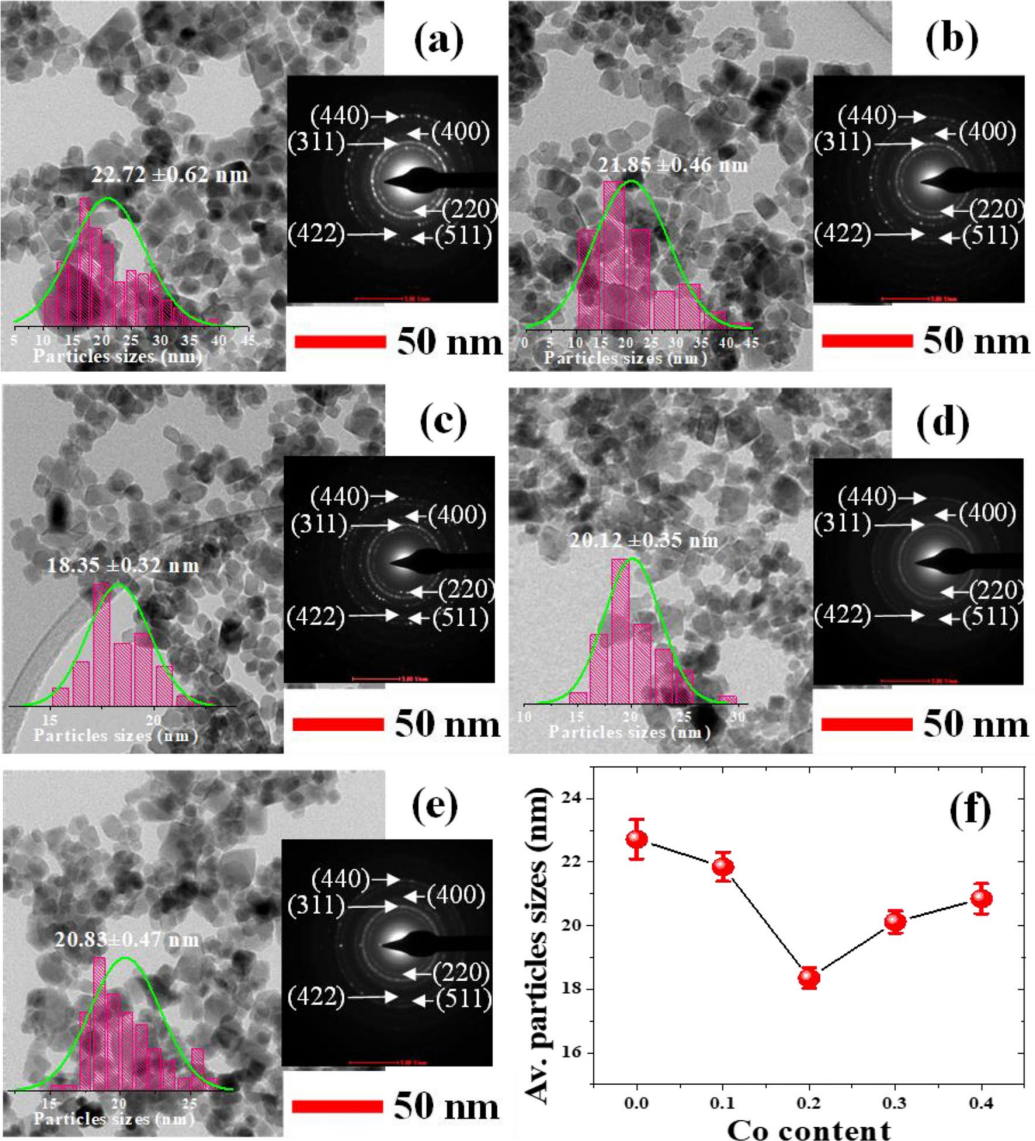
TEM images with SAED patterns and histograms showing the particle size distribution of Co_x_Zn_1−x_Fe_2_O_4_ NPs (**a**) x = 0.0, (**b**) x = 0.1, (**c**) x = 0.2, (**d**) x = 0.3, and (**e**) x = 0.4. (**f**) Plot of Av. particles sizes as a function of Co content.

TEM images of Co_x_Zn_1−x_Fe_2_O_4_ (x = 0.0–0.4) NPs with insets displaying selected area electron diffraction (SAED) patterns and the histograms of estimated Av. particle size are shown in Fig. 6a–e. In these figures, NPs of a cuboidal shape with a uniform dispersion of particle sizes over a narrow range are observed, which is also seen in the SEM images. In the TEM images, the Av. particle sizes of all samples could be more precisely measured using the Image J program. A plot of the obtained values as a function of Co content is shown in Fig. 6f. In this figure, the average particle size decreases with Co loading, corresponding to the SEM results. However, the decreased particle sizes of Co_1−x_Zn_x_Fe_2_O_4_ to its lowest value for x = 0.2 and then its increase with Co content (for x = 0.3 and 0.4), as shown in Figs. 5f and 6f, might be dependent on the different growth rates. Additionally, Co cations might be substituted at A and B sites in different amounts during the preparation process. Additionally, all the observed SAED patterns display halo rings with bright spots on their circumferences. These rings can be indexed to the (220), (311), (400), (422), (511) and (440) planes of a ZnFe_2_O_4_ phase, indicating the polycrystalline nature of these materials. Moreover, these indexed diffraction rings have been verified to correspond with a Fd-3m space group, agreeing well with the XRD results and the study of Mohamed et al*.*^18^. From the XRD, SEM and TEM results, Co doping can significantly reduce the crystallite and particle sizes of Co_x_Zn_1−x_Fe_2_O_4_ (x = 0.0ؘ–0.4) NPs.

### FTIR study

**Figure 7:**
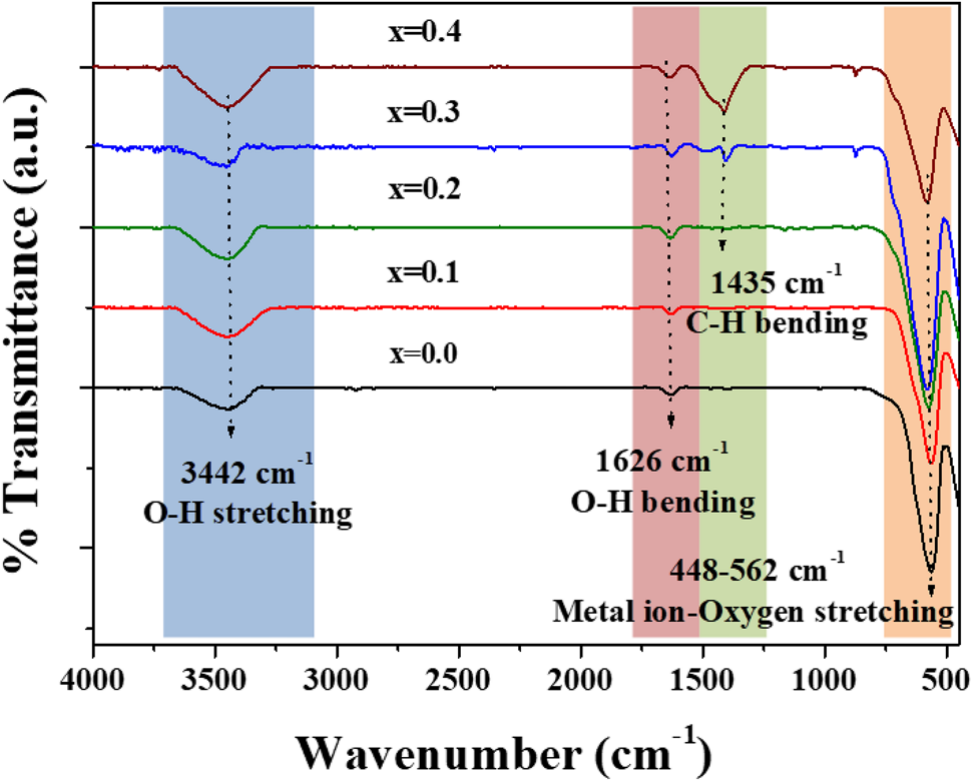
FTIR spectra of Co_x_Zn_1−x_Fe_2_O_4_ (x = 0.0–0.4) NPs.

FTIR measurements were performed over a wide range of wavenumbers from 400 to 4000 cm^−1^. The obtained spectra are displayed in Fig. 7. As illustrated in this figure, all spectra are similar. The observed spectra at around 3442 and 1626 cm^−1^ correspond to O–H stretching and bending modes, respectively. This may be due to the absorption of water molecules by KBr pellets during spectroscopic studies. In the case of Co_0.3_Zn_0.7_Fe_2_O_4_ and Co_0.4_Zn_0.6_Fe_2_O_4_ NPs, the peak at 1626 cm^−1^ shifts to a slightly lower frequency upon substitution of Co, implying that both samples are sensitive to moisture and water molecules^29^. Additionally, the peak at 1435 cm^−1^ corresponds to the bending modes of anionic carboxylate C-H by ethylene glycol (EG) coated on the surfaces of nanoparticles^30,31^. EG can function as a stabilizer that uses surfactant molecules to form coordination bonding or strong interaction. This can prevent aggregation of mixed spinel Co_0.3_Zn_0.7_Fe_2_O_4_ and Co_0.4_Zn_0.6_Fe_2_O_4_ NPs. Moreover, it can kinetically control the growth rates of crystals and the morphology of NPs, as reported by Gözüak et al.^25^. Additionally, it is established that small particle sizes and good dispersion are helpful for electrolyte ions to penetrate the porous surfaces of the electrodes and to provide for high specific capacitance. The spectra at 448 to 562 cm^−1^ were attributed to the characteristic vibrational modes of metals ion and oxygen atoms in the spinel structure^32–34^. Specifically, the wavenumber at 448 cm^−1^ was ascribed to the stretching mode of metal and oxygen atoms at the B site (octahedral site). However, the same vibration mode at 562 cm^−1^ was attributed to the stretching mode of metal and oxygen atoms at the A site (tetrahedral site). Moreover, as the Co content increases, the peak position of vibrational mode at A site slightly shifts to a higher frequency region due to displacement of a heavy atom (Zn) by a lighter atom (Co)^18,35^.

### Oxidation state analysis

**Figure 8:**
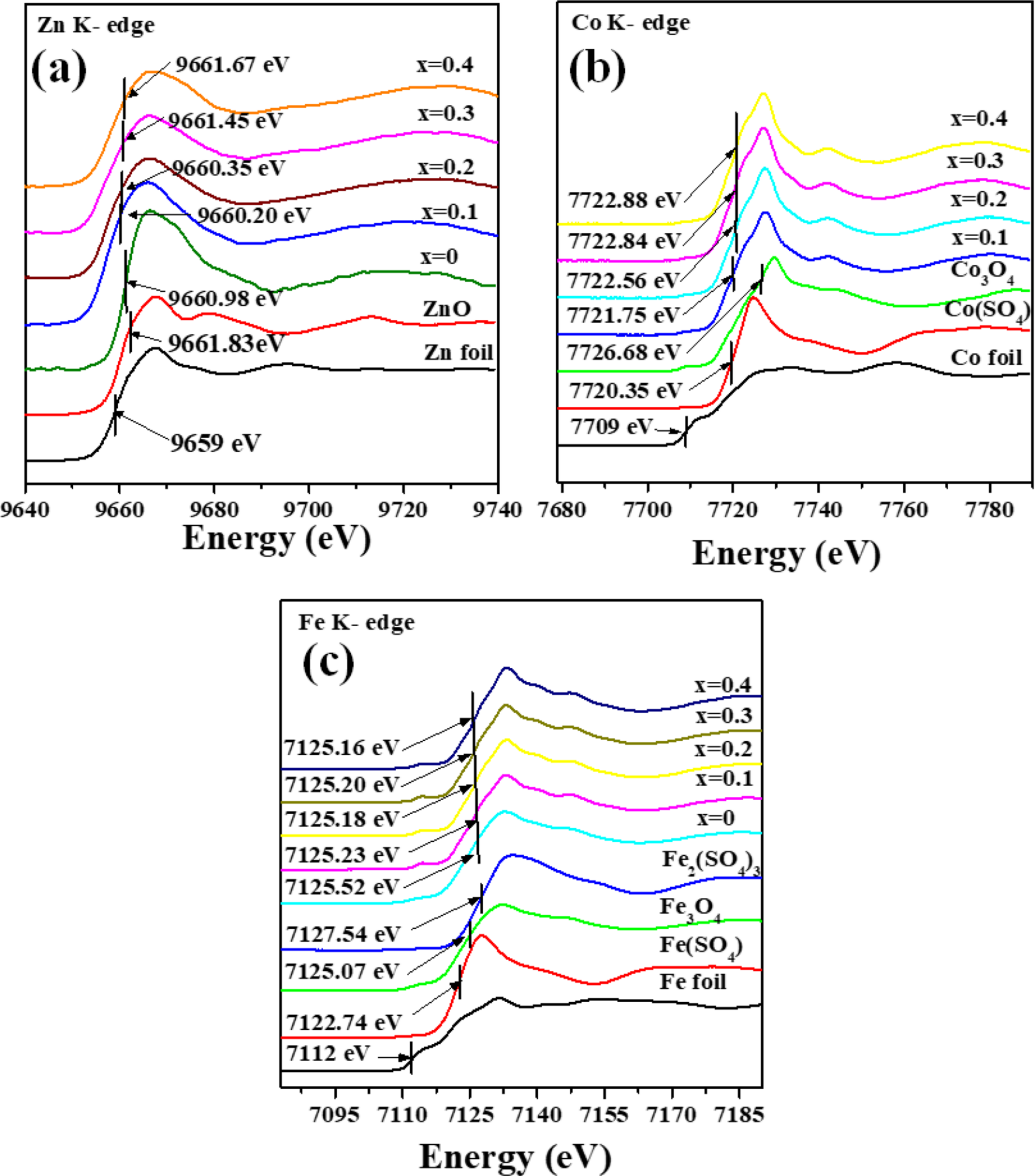
(**a**) Zn, (**b**) Co and (**c**) Fe K-edge XANES spectra of various standard materials and Co_x_Zn_1−x_Fe_2_O_4_ (x = 0.0–0.4) NPs.

Figure 8a displays the normalized Zn K-edge XANES spectra of Co_x_Zn_1−x_Fe_2_O_4_ (x = 0.0–0.4) NPs, as well as Zn foil and ZnO standards. As seen in Fig. 8a, the Zn foil and ZnO (having oxidation states of 0 and + 2, respectively) show the maximum gradient of their spectra at different energy positions, 9659.0 and 9661.83 eV, respectively^36–38^. For our samples, all the spectra display their maximal gradients in the range of 9660.20–9661.67 eV, implying that the oxidation state of the Zn cation is + 2, in agreement with the work of Wiriya et al*.*^37^. In Fig. 8b, the Co foil, Co(SO_4_), and Co_3_O_4_ standards reveal, respectively, the maximum gradient Co K-edge XANES spectra at energy positions of 7709, 7720.35, and 7726.68 eV. These energy positions correspond to the oxidation states of 0, + 2, and (+ 2, + 3), respectively. Similarly, our samples (x = 0.1–0.4) reveal their maximum gradient Co K-edge XANES spectra at energy positions of 7721.75, 7722.56, 7722.84 and 7722.88 eV, respectively, which are close to that of Co(SO_4_). Therefore, it is suggested that the oxidation state of the Co cation is + 2 with redistribution of the ions at A- and B-sites in the spinel structure^39,40^. In Fig. 8c, the maximum gradient Fe K-edge XANES spectra of Fe foil, Fe(SO_4_), Fe_3_O_4_, and Fe_2_(SO_4_)_3_ appear, respectively, at 7112, 7122.74, 7125.07 and 7127.54 eV, corresponding to the oxidation states of 0, + 2, (+ 2, + 3) and + 3, respectively. In the case of our samples with x = 0.0, 0.1, 0.2, 0.3 and 0.4, the maximum gradient Fe K-edge spectra appear at energy position of 7125.52, 7125.23, 7125.18, 7125.20 and 7125.16 eV, respectively, suggesting oxidation states + 2 and + 3 of Fe cations for these samples. Regarding our results and others related to the studies of Nilmoung et al*.*^2^, Wiriya et al*.*^37,40^ and Liang et al*.*^41^, Co^2+^ ions can replace Zn^2+^ ions at both the A and B sites, while Fe^2+^ and Fe^3+^ ions can be distributed at both sites of a mixed spinel Co_x_Zn_1−x_Fe_2_O_4_ (x = 0.1–0.4) NPs, as shown in Fig. 3d. Additionally, the probable distribution of these ions at A and B sites is proposed as the following:

- [A] site [B] site
- Normal spinel ZnFe_2_O_4_ [Zn^2+^] [Fe^2+^/Fe^3+^]O_4_^2-^
- Inverse spinel CoFe_2_O_4_ [Fe^2+^/Fe^3+^] [Co^2+^, Fe^2+^/Fe^3+^]O_4_^2-^
- Mixed spinel Co_x_Zn_1−x_Fe_2_O_4_ [(Co_x_^2+^, Zn_1−x_^2+^)_y_, Fe^2+^/Fe^3+^] [(Co_x_^2+^, Zn_1−x_^2+^)_1−y_, Fe^2+^/Fe^3+^]O_4_^2−^

### Surface area study

**Figure 9:**
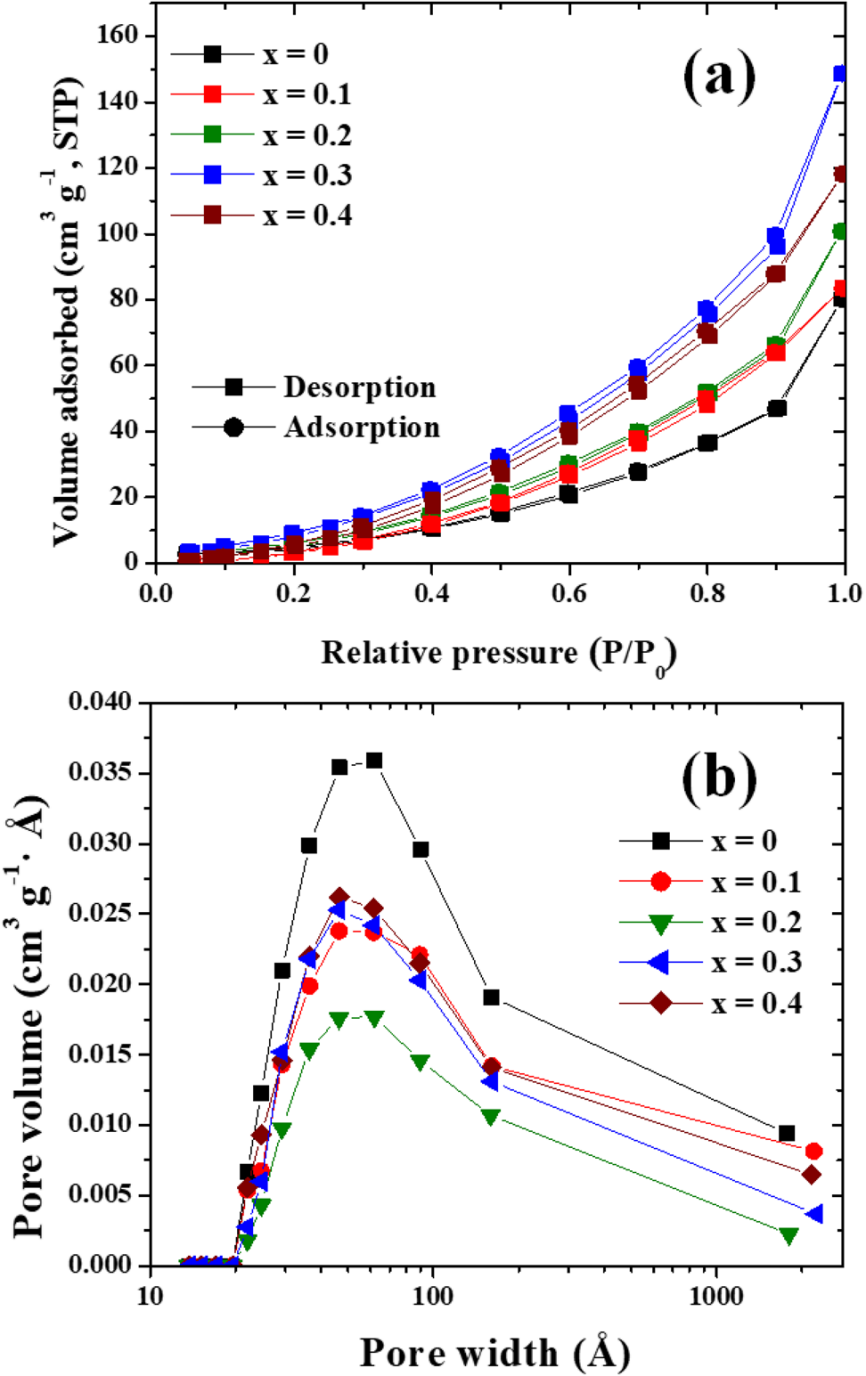
(**a**) BET isotherm, (**b**) pore size distribution curves of Co_x_Zn_1−x_Fe_2_O_4_ (x = 0.0–0.4) NPs.

Figure 9a displays N_2_ adsorption–desorption isotherms of Co_x_Zn_1−x_Fe_2_O_4_ (x = 0.0–0.4) NPs. These results were found to be typical type IV isotherms with small hysteresis loops, representing a mesoporous nature^1^. Clearly, Co_0.3_Zn_0.7_Fe_2_O_4_ (x = 0.3) NPs exhibit the maximum BET surface area, 84.30 m^2^ g^−1^. Other electrodes, with x = 0.0, 0.1, 0.2 and 0.4, show lower surface areas, 37.05, 58.84, 64.38 and 75.31 m^2^ g^−1^, respectively. Based on the BJH model and the plot of pore volume *vs.* pore width shown in Fig. 9b, the pore volumes of Co_x_Zn_1−x_Fe_2_O_4_ (x = 0.0–0.4) NPs were calculated and found to be 0.01833, 0.02491, 0.2306, 0.03127 and 0.0322 cm^3^/g, respectively. Moreover, the pore size of Co_x_Zn_1−x_Fe_2_O_4_ (x = 0.0–0.4) NPs were determined to be 16.93, 16.90, 14.33, 14.84 and 17.11 nm, respectively. It is clear that Co doping can significantly impact the surface area, pore volume and pore size of Co_x_Zn_1−x_Fe_2_O_4_ (x = 0.1–0.4) NPs, and gives evidence of increased active sites and shortened transport paths at electrodes surfaces^7^.

### Electrochemical properties study

**Figure 10:**
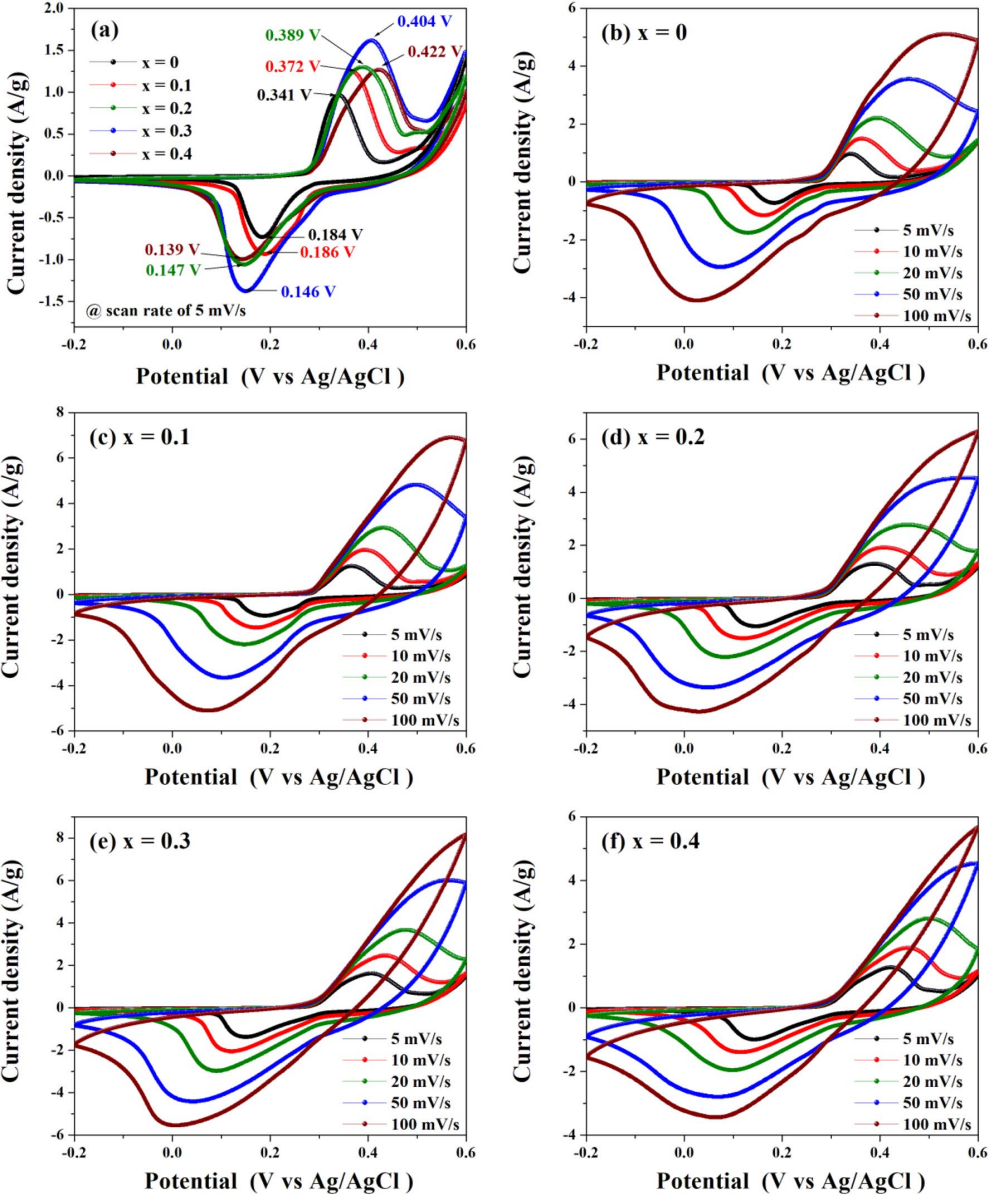
CV curves of Co_x_Zn_1−x_Fe_2_O_4_ (x = 0.0–0.4) NPs electrodes measured at scan rate (**a**) 5 mV/s and (**b**)–(**f**) 5–100 mV/s.

The CV results of Co_x_Zn_1−x_Fe_2_O_4_ (x = 0.0–0.4) NPs electrodes recorded at a scan rate 5 mV/s over a potential window −0.2 to 0.6 V are shown in Fig. 10a at different scan rates of 5–100 mV/s in Fig. 10b–f. In Fig. 10a, a pair of reversible redox or faradaic redox reaction peaks can be observed in every curve, indicating a pseudocapacitive behavior of these electrodes^1,5,15,19,42^. For the ZnFe_2_O_4_ (x = 0) electrode, anodic and cathodic peaks appear at around 0.341 V and 0.184 V, respectively, which can be ascribed to stepwise oxidation and reduction of Zn^2+^ and Fe^3+^/Fe^2+^ in a KOH electrolyte, similar to earlier literature reports^10,15,42^. The charging process in ZnFe_2_O_4_ normal spinel is due to coupling between Zn^2+^ at its A site and Fe^3+^/Fe^2+^ at the B site. During discharge, Fe^3+^/Fe^2+^ and Zn^2+^ can become distributed among both sites, introducing a reversible redox reaction. However, in Co_x_Zn_1−x_Fe_2_O_4_ (x = 0.1–0.4) NPs electrodes, the anodic peaks were shifted from 0.372 to 0.422 V, while the cathodic peaks ranged from 0.186 to 0.139 V with increasing Co content. These anodic and cathodic peaks are related to the Co^2+^ and Zn^2+^ ion occupancy at the B site and Fe^3+^/Fe^2+^ at both A and B sites. This is a result of redox couples with the assistance of OH^−^ in the KOH electrolyte^42,43^. Furthermore, it is observed that Co_0.3_Zn_0.7_Fe_2_O_4_ (x = 0.3) electrode shows the largest CV area, corresponding to the highest *Csc* value among these products. Figure 10b–f show CV curves the Co_x_Zn_1−x_Fe_2_O_4_ NPs electrodes performed at various scan rates from 5–100 mV/s. With increasing Co content and scan rate, the anodic and cathodic peaks reveal increased current values and areas of redox peaks. Optimal values were observed in the x = 0.3 electrode, indicating that the charge storage process is dominated by a diffusion-controlled redox reaction^5,43^. Moreover, the anodic and cathodic peaks at all potential values of every electrode were slightly shifted towards higher and lower potentials, respectively. This might be caused by a polarization effect due to an increased Co content. As seen in Fig. 10e, the closed CV curve of the Co_0.3_Zn_0.7_Fe_2_O_4_ (x = 0.3) electrode is larger than those of other electrodes at different sweep rates, implying the best electrochemical performance.

**Figure 11:**
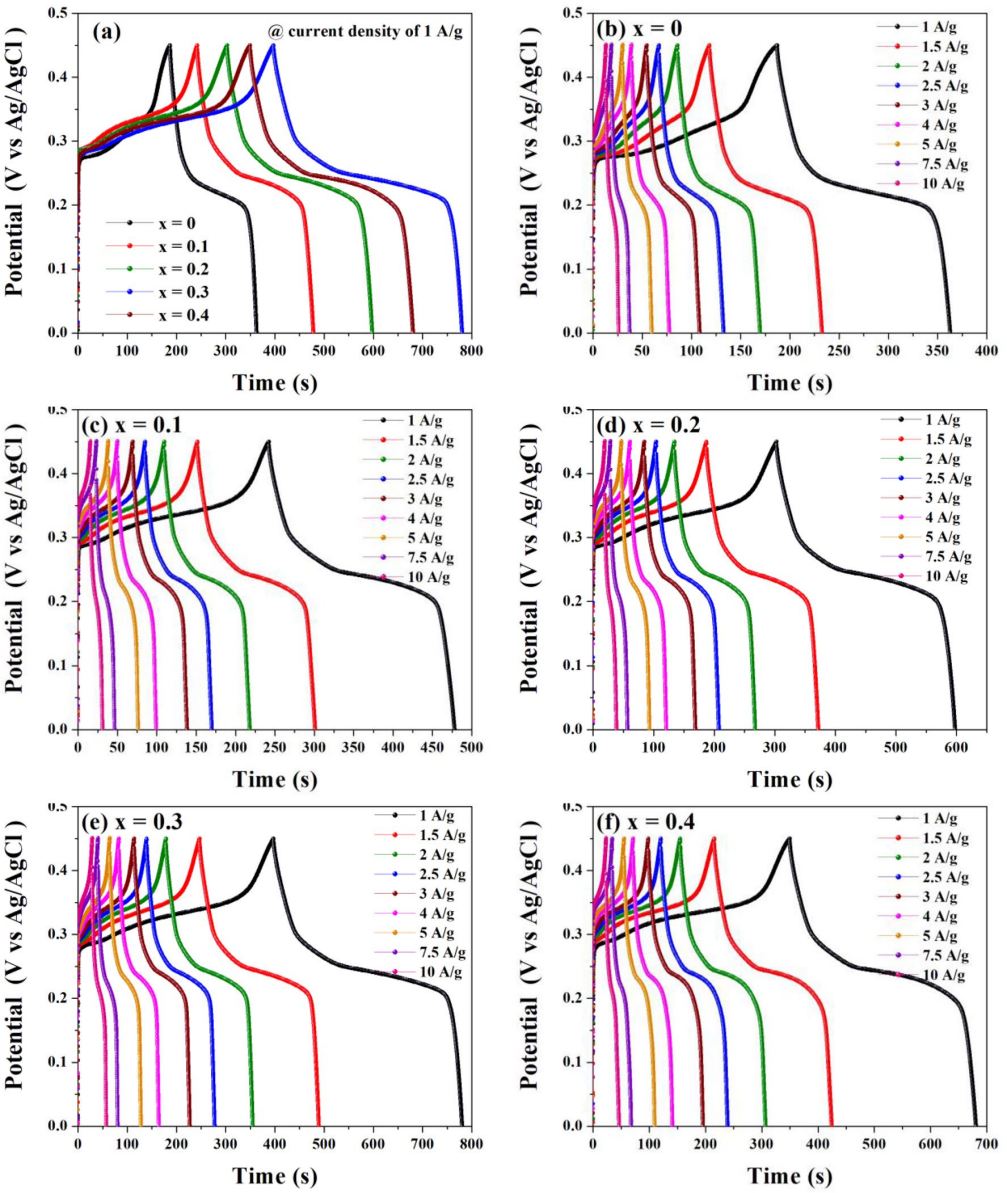
GCD curves of Co_x_Zn_1−x_Fe_2_O_4_ (x = 0.0–0.4) NPs electrodes measured at various current densities (**a**) 1 A/g and (**b**)–(**f**) 1–10 A/g.

The GCD results measured over a potential window 0–0.45 V at 1 A/g and various current densities of 1–10 A/g were used to determine the capacitive performance of Co_x_Zn_1−x_Fe_2_O_4_ (x = 0.0–0.4) NPs electrodes, as shown in Fig. 11a–f. In these figures, all GCD curves show a relatively asymmetric shape, implying a favorable pseudocapacitive behavior^8,19,44^ for all electrodes. The GCD results of the Co_0.3_Zn_0.7_Fe_2_O_4_ (x = 0.3) electrode show the highest specific capacitance among these products, agreeing well with the CV results. Moreover, the GCD curves of all Co_x_Zn_1−x_Fe_2_O_4_ NPs electrodes display a very low internal resistance, suggesting good conductivity of electrode materials^4,15,21^.

**Figure 12:**
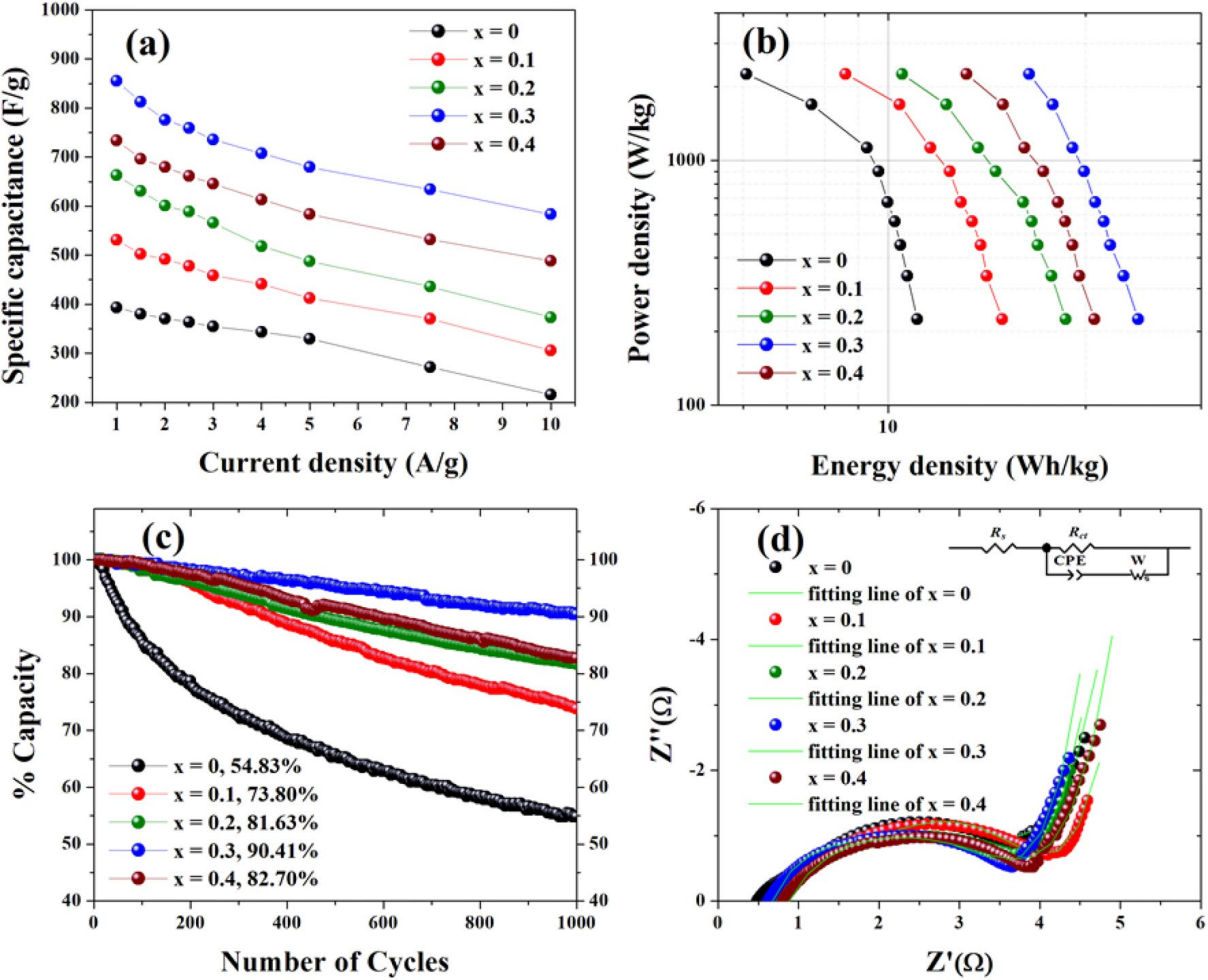
(**a**) Plots of specific capacitance as a function of current density, (**b**) Ragone plots at various current densities, (**c**) retention after 1000 cycles during charge–discharge testing at a current density of 5 A/g and (**d**) line fitting of the Nyquist plots of Co_x_Zn_1−x_Fe_2_O_4_ (x = 0.0–0.4) NPs electrodes.

Furthermore, the specific capacitances (*Csc*) of Co_x_Zn_1−x_Fe_2_O_4_ (x = 0.0–0.4) NPs electrodes were calculated with Eq. (1), using the integral discharge curves at various current densities from 1–10 A/g. The obtained data are plotted in Fig. 12a and summarized in Table 2. In this figure, the *Csc* values decease with increasing current density due to the decay of electrodes during redox reactions at high current density^3,15^. Clearly, the Co_0.3_Zn_0.7_Fe_2_O_4_ (x = 0.3) electrode exhibits the maximum *Csc* value, 855.33 F/g, at a current density of 1 A/g, while other electrodes with x = 0.0, 0.1, 0.2 and 0.4 show lower *Csc* values of 391.51, 530.86, 662.75 and 733.53 F/g, respectively. Figure 12b shows a Ragone plot of Co_x_Zn_1−x_Fe_2_O_4_ (x = 0.0–0.4) NPs electrodes over a potential window of 0.0–0.45 V and at various current densities of 1–10 A/g in a KOH electrolyte. It is clearly seen in Fig. 12b that the Co_0.3_Zn_0.7_Fe_2_O_4_ (x = 0.3) electrode delivers the highest energy density. This enhanced electrochemical performance is suggested to arise from improved electroactive sites with shortened conduction pathways, as well as numerous nanopaths that promote transport of various ionic species due to the presence of Co^2+^ ions in the structure^21^. Furthermore, Fig. 12c shows the capacity retention of Co_x_Zn_1−x_Fe_2_O_4_ (x = 0.0–0.4) NPs electrodes after 1000 GCD cycle testing at 5 A g^−1^. The capacity retention was found to be 54.83, 73.80, 81.63, 90.41 and 82.70% for Co_x_Zn_1−x_Fe_2_O_4_ electrodes with x = 0.0, 0.1, 0.2, 0.3 and 0.4, respectively. As can be seen, the Co_0.3_Zn_0.7_Fe_2_O_4_ (x = 0.3) electrode exhibits better cycling stability among the electrodes. It is suggested that this might be due to more effective contact of the Co_0.3_Zn_0.7_Fe_2_O_4_ electrode and KOH electrolyte. This promotes simultaneous faster ion/charge transport in the bulk of electrode and at the electrode/electrolyte interface to these achieve excellent properties^6,8,12,38^. Moreover, this electrode has a higher active area via a uniform distribution of nanosized particles that prevents particle agglomeration, as shown in the SEM, TEM, and BET results. This enhances the electrical conductivity of the electrode^10^. The highest *Csc* value obtained in the Co_0.3_Zn_0.7_Fe_2_O_4_ (x = 0.3) electrode is superior to values reported in the literature on spinel TMOs (AB_2_O_4_), as illustrated in Table 3.

**Table 2:** Specific capacitance at various current densities, capacity retention after 1000 GCD cycle testing at 5 A/g and EIS analysis of Co_x_Zn_1−x_Fe_2_O_4_ (x = 0.0–0.4) NPs electrodes.

Parameter		ZnFe_2_O_4_ NP	Co_x_Zn_1−x_Fe_2_O_4_ NPs			
			x = 0.1	x = 0.2	x = 0.3	x = 0.4
*Csc* (F/g)	1 A/g	393.51	530.86	662.75	855.33	733.53
	1.5 A/g	380.26	502.36	631.20	812.67	696.07
	2 A/g	370.80	492.13	601.06	775.56	679.42
	2.5 A/g	363.77	477.56	588.72	758.89	661.67
	3 A/g	354.53	458.67	565.93	735.67	645.53
	4 A/g	343.55	441.24	518.48	707.64	613.42
	5 A/g	329.78	412.12	487.66	679.45	583.56
	7.5 A/g	271.33	370.16	435.83	634.01	532.22
	10 A/g	215.70	306.05	373.10	583.56	488.67
Capacity retention (%) at 5 A/g		54.83	73.80	81.63	90.41	82.70
EIS analysis	Rs (Ω)	0.55	0.67	0.71	0.68	0.82
	Rct (Ω)	4.56	4.46	3.83	3.63	3.72
	Rw	4.08	5.31	6.75	7.32	5.60

**Table 3:** Electrochemical properties of electrodes fabricated from various spinel TMOs (AB_2_O_4_) and synthesized Co_0.3_Zn_0.7_Fe_2_O_4_ NPs electrodes.

Electrode material	Synthesis method (Morphology)	*Csc* value at current density or scan rate	capacity retention at cycles	electrolyte
CoFe_2_O_4_^1^	Co-precipitation method (Nanoparticles)	1210 F/g at 1 A/g	82% at 5000	1 M KOH
NiFe_2_O_4_@CoFe_2_O_4_^3^	Electrospinning method (Nanofibers)	480 F/g at 1 A/g	87% at 2000	3 M KOH
MnFe_2_O_4_^4^	Hydrothermal method (Nanoparticles)	1221 F/g at 0.5 A/g	90% at 1500	3 M KOH
Ni_0.4_Co_0.6_Fe_2_O_4_^5^	Wet chemical method (Nanoparticles)	237 F/g at 1 A/g	97% at 4000	3 M KOH
CoFe_2_O_4_/NiFe_2_O_4_^6^	Hydrothermal method (Nanospheres)	269 F/g at 1 A/g	81% at 1000	2 M KOH
ZnFe_2_O_4_^11^	Solvothermal technique (Microspheres)	175 F/g at 5 A/g	82.75% at 1000	1 M KOH
ZnFe_2_O_4_^12^	Wet chemical method (Nanospheres)	615 F/g at 3 mA/cm^2^	–	6 M KOH
ZnFe_2_O_4_/NRG^13^	Solvothermal method (Nanocrystals)	244 F/g at 0.5 A/g	83.8% at 5000	1 M KOH
ZnFe_2_O_4_^15^	Sonochemical method (Nanoparticles)	712 F/g at 2 mV/s	96.6% at 2000	6 M KOH
CoFe_2_O_4_/MWCNTs^19^	Sonochemical synthesis (Nanocomposite)	390 F/g at 1 mA/cm^2^	86.9% at 2000	1 M KOH
Ce_0.3_CoFe_1.7_ O_4_^20^	Hydrothermal method (Nanostructure)	625 F/g at 0.5 A/g	–	1 M KOH
CoFe_2_O_4_/CNTs^21^	Hydrothermal method (Nanocomposite)	1240 F/g at 0.5 A/g	75.8% at 1000	2 M KOH
CoFe_2_O_4_^44^	solution combustion method (Nanoparticles)	195 F/g at 1 mV/s	–	1 M KOH
CoFe_2_O_4_/rGO^45^	Solvothermal method (Nanocomposite)	551 F/g at 2 mV/s	98% at 2000	2 M KOH
Co_0.3_Zn_0.7_Fe_2_O_4_^This work^	Hydrothermal method (Nanoparticles)	855.33 F/g at 1 A/g	90.41% at 1000	3 M KOH

The highest *Csc* value obtained in Co_0.3_Zn_0.7_Fe_2_O_4_ (x = 0.3) electrode is superior to earlier related work reported in literature on spinel TMOs (AB_2_O_4_), as illustrated in Table 3. Clearly in Table 3, the Co_0.3_Zn_0.7_Fe_2_O_4_ (x = 0.3) electrode prepared by a hydrothermal method, shows a higher *Csc* value than those obtained in many of TMOs (AB_2_O_4_) ferrites. The high specific capacitance of the Co_0.3_Zn_0.7_Fe_2_O_4_ (x = 0.3) electrode might be due to a homogeneous distribution of nanosized particles and the mesoporous nature of the material, which could promote electrolyte access. Moreover, with the high cycling stability retention of Co_0.3_Zn_0.7_Fe_2_O_4_ (x = 0.3) electrode, this material is a good candidate for supercapacitor applications. It is remarkable that Co ions in Co-doped ZnFe_2_O_4_ NPs can play a significant role in improving the electrochemical properties of these materials. Co ions can influence the interstitial sites of a ZnFe_2_O_4_ lattice, which can enhance the electrochemical activities of the electrode material. Moreover, the morphology of nanosized cuboidal shape-particles with good dispersion are supportive of electrolyte ion penetration of the porous electrode surface. This could result in a high specific capacitance. Additionally, Co doping could possibly increase cation transfer (Co^2+^, Zn^2+^ and Fe^3+^/Fe^2+^) between A and B sites, resulting in more redox couples with the assistance of OH^−^ in KOH electrolyte.

Figure 12d shows the EIS spectra of Co_x_Zn_1−x_Fe_2_O_4_ (x = 0.0–0.4) NPs electrodes, where the plots of the real part (Z') *vs.* an imaginary part (Z'') were obtained of a frequency range of 0.01 Hz to 10 MHz using an applied AC voltage of 10 mV. The EIS analysis data for each electrode is given in Table 2. Additionally, the Randle’s equivalent circuit employed for EIS analysis, primarily consisting of the solution resistance (Rs), charge transfer resistance (Rct), constant phase element (CPE) and Warburg resistance (Rw), is displayed in the inset of Fig. 12d. The plots shown in Fig. 12d display three regions, depending on the applied frequency^1,7^. Initially, the Z' axis intercept in the high frequency range represents the solution resistance (Rs) of the 3 M KOH electrolyte and working electrode interface^6,15,38,43^. The Rs value for each Co_x_Zn_1−x_Fe_2_O_4_ (x = 0.0, 0.1, 0.2, 0.3 and 0.4) NPs electrode was found to be 0.55, 0.67, 0.71, 0.68 and 0.82 Ω, respectively. However, the slight increase of Rs causes a reduction in the conductivity of the KOH electrolyte^6,38^. In the next frequency range, where semicircular loops are observed, the diameter of each semicircular loop is related to electron charge transfer resistance (Rct) at the electrode and electrolyte interface, resulting from the Faradaic redox process^6,38,43,44^. Accordingly, the Rct values were respectively found to be 4.56, 4.46, 3.83, 3.63 and 3.72 Ω for Co_x_Zn_1−x_Fe_2_O_4_ (x = 0.0, 0.1, 0.2, 0.3 and 0.4) NPs electrodes. It is clear that the Rct value of the Co_0.3_Zn_0.7_Fe_2_O_4_ (x = 0.3) electrode is lower than that of other electrodes, indicating higher electrolytic ion diffusion. This might be due to the homogeneous surface of electrode that could provide area for effective active sites for the OH^−^ ions of the KOH electrolyte, promoting their easy access. This is in good agreement with the lowest Rct value^15,42,43^. Last, in the low frequency range, the observed slope of the straight-line portion of the curve reflects the Warburg resistance (Rw). This represents the ion diffusion process of redox material in the KOH electrolyte^6,38^. The increased slope in this frequency range indicates that the materials have become more purely capacitive^2,32^. The slope values of Co_x_Zn_1−x_Fe_2_O_4_ (x = 0.0, 0.1, 0.2, 0.3 and 0.4) NPs electrodes were determined to be 4.08, 5.31, 6.75, 7.32 and 5.60, respectively. As seen, the slope value of the Co_0.3_Zn_0.7_Fe_2_O_4_ (x = 0.3) electrode is highest among the electrodes, suggesting its faster ion transfer from the electrolyte and providing a greater *Csc* value^7,44^.

## Conclusion

Co_x_Zn_1−x_Fe_2_O_4_ (x = 0.0–0.4) NPs samples were successfully synthesized using a hydrothermal method. All Co_x_Zn_1−x_Fe_2_O_4_ NPs have a cubic spinel structure as revealed by XRD. The *a*, *D*_XRD_ and Av. particles sizes of Co_x_Zn_1−x_Fe_2_O_4_ NPs slightly decreased with increasing Co content (8.402 to 8.353 Å for* a*, 19.81 ± 4.8 to 14.23 ± 2.9 nm for* D*_XRD_ and 22.72 ± 0.62 to 20.85 ± 0.47 nm for the Av. particles sizes). This was due to the substitution of Co^2+^ ions of smaller ionic radius at the A and B sites in a suggested mixed spinel structure of Co_x_Zn_1−x_Fe_2_O_4_ NPs. SEM and TEM images displayed the homogeneous distribution of NPs of a cuboidal shape, which could significantly increase the active surface area of electrodes (supported by the BET results) and beneficially provide optimal penetration paths for fast ion/electron transfer. The results in electrochemical enhancement of electrodes. The presence of Zn^2+^, Co^2+^ and Fe^2+^ and Fe^3+^ ions in all samples was confirmed by the XANES results. According to the suggested mixed spinel ferrites of Co_x_Zn_1−x_Fe_2_O_4_ NPs, Co^2+^ could replace Zn^2+^ ions at A and B sites, while Fe^2+^ and Fe^3+^ ions could be redistributed at both sites as well. The distribution of these ions could well provide for the Faradaic redox reactions of a pseudo-capacitive characteristic of the materials, as illustrated by the CV, GCD and EIS results. From these results, it is emphasized that Co ions could significantly influence the structure, morphology and electrochemical properties of Co_x_Zn_1−x_Fe_2_O_4_ NPs. The highest *Csc* value, 855.33 F/g at 1 A/g with a 90.41% capacity retention after 1000 GCD cycle testing, was achieved in a working Co_0.3_Zn_0.7_Fe_2_O_4_ (x = 0.3) NPs electrode. Based on these results, the Co_0.3_Zn_0.7_Fe_2_O_4_ NPs are suggested as a suitable candidate material for supercapacitor electrodes.

## Data Availability

All data generated or analyzed during this study are included in this published article.
